# Beyond the Limits: Severe Hyperglycemia in Hyperosmolar Hyperglycemic State (Serum Glucose 2375 mg/dL)

**DOI:** 10.1016/j.aed.2025.11.013

**Published:** 2025-12-13

**Authors:** Karthik Iyer, Riley Bricker, Aanika Gupta, Moye Mathew, Robert Hieger, Shrenika Reddy, Ananya Ganesan, Theodore Rogers, Vikram Oke

**Affiliations:** 1 Division of Critical Care Medicine, Department of Internal Medicine, Mercy Hospital Jefferson, Festus, Missouri; 2 Lake Erie College of Osteopathic Medicine, Erie, Pennsylvania; 3 Department of Internal Medicine, Mercy Hospital Jefferson, Festus, Missouri; 4 Department of Family Medicine, Mercy Hospital Jefferson, Festus, Missouri; 5 Division of Endocrinology, Department of Internal Medicine, Mercy Hospital Jefferson, Festus, Missouri; 6 Department of Family Medicine, Mercy Hospital Perry, Perryville, Missouri

**Keywords:** HHS, hyperosmolar hyperglycemic state, severe hyperglycemia, type 1 diabetes mellitus

## Abstract

**Background/Objective:**

Hyperosmolar hyperglycemic state (HHS) is a life-threatening diabetic emergency characterized by extreme hyperglycemia, dehydration, and absence of significant ketoacidosis. This case is noteworthy due to the patient’s extraordinarily high serum glucose level of 2375 mg/dL, one of the highest recorded in literature. This report's objective is to describe a patient with HHS and profound hyperglycemia that challenges the known boundaries of human physiology.

**Case Presentation:**

A 28-year-old transgender man (assigned female at birth) receiving testosterone replacement therapy presented to a critical access hospital emergency department with severe nausea, vomiting, and signs of dehydration. He had insulin-dependent type I diabetes mellitus and stage 3 chronic kidney disease. Initial laboratory testing revealed serum glucose of 2375 mg/dL (reference range: 70-140 mg/dL). Laboratory analysis showed no ketoacidosis. He was diagnosed with a hyperosmolar hyperglycemic state. The patient was transferred to our hospital and treated with intravenous fluids and insulin. Laboratory values were closely monitored. Over several days, glucose levels declined to reference ranges. His symptoms resolved, and he was discharged in a stable condition.

**Discussion:**

HHS typically occurs in older adults with type 2 diabetes and is associated with infections or organ failure. Serum glucose above 2000 mg/dL is rarely reported. This case stands out due to the significant level of hyperglycemia.

**Conclusion:**

This case contributes to limited literature on profound hyperglycemia and emphasizes the importance of early recognition and treatment.

**Highlights**

• Hyperosmolar hyperglycemic state (HHS) can rarely present with serum glucose greater than 2000 mg/dL, which is life-threatening. Serum glucose of 2375 mg/dL is among the highest ever reported
• The absence of significant ketosis distinguishes HHS from diabetic ketoacidosis, as residual insulin suppresses ketone formation despite extreme hyperglycemia
• Profound dehydration is the hallmark of HHS, and aggressive fluid resuscitation before insulin therapy remains the cornerstone of management
• HHS typically affects older adults with type 2 diabetes, though it may be the initial presentation of previously undiagnosed diabetes

**Clinical Relevance**

This case demonstrates survival after one of the highest recorded serum glucose levels and highlights the importance of early recognition, rapid fluid resuscitation, and timely insulin therapy, even in resource-limited or rural settings.

## Introduction

In the 1800s, hyperosmolar hyperglycemic state (HHS) was first described in patients with glycosuria and hyperglycemia but without typical diabetic ketoacidosis (DKA) features such as fruity breath, urinary acetone, or Kussmaul breathing. Subsequently, similar cases of well-nourished adults falling into diabetic coma were reported.^1^

Type 1 diabetes mellitus (T1DM) results from autoimmune destruction of pancreatic B-cells, causing insulin deficiency and hyperglycemia. Its incidence in adolescents and related crises like DKA and HHS have increased.^1^

Type II diabetes mellitus (T2DM) is characterized by insulin resistance.^2^ Early in the disease, pancreatic beta cells compensate by increasing insulin production, but over time, this fails, leading to hyperglycemia.^2^ Altered fat metabolism and hepatic glucose production further worsen the condition. Once common in adults over 45, rising obesity and sedentary lifestyles have increased T2DM cases in youth.^3^

HHS is often seen with a lack of functional insulin, as seen in diabetes, and poor fluid intake.^2^ The increase in blood glucose secondary to the lack of functional insulin induces osmotic diuresis, thus causing further fluid volume loss.^2^ HHS initially presents with slowly increasing polyuria and polydipsia that progresses to significant dehydration and electrolyte loss.^1^ Additional symptoms such as nausea, weakness, and behavioral changes can be attributed to signs of volume depletion and electrolyte loss.^4^ One of the major distinguishing factors between DKA and HHS is the presence or lack of ketonemia. HHS does not classically present with ketonemia.^2^ This lack of ketosis is likely because patients with T2DM do not have a complete lack of insulin; therefore, the production of ketones is suppressed by the presence of insulin.^2^ Importantly, this lack of ketosis does not equate to a less severe disease, as both ailments can prove life-threatening.

## Case Description

A 28-year-old transgender male presented to a critical access hospital emergency department (ED) with persistent nausea and vomiting for 48 h. On arrival, he was fully oriented, appropriately answering questions, and able to follow commands. He reported worsening symptoms and new-onset polydipsia, describing intense cravings for water and ice chips. Shortly thereafter, his neurological status declined rapidly, and he became confused and unable to follow commands.

The patient’s past medical history included insulin dependent T1DM, chronic kidney disease stage 3b, hiatal hernia, gastroparesis, unspecified bipolar disorder, generalized anxiety disorder, and autism. Past surgical history includes cholecystectomy, esophageal dilation, deep neck lymph node biopsy/excision, tonsillectomy, and adenoidectomy. No family history of diabetes was reported. He endorsed former tobacco use and reported occasional marijuana use.

A review of current medications indicated the use of an insulin pump and continuous glucose monitor for several months, with the only notable event being transitioning to a basal insulin bolus strategy when the pump becomes empty. Other medications include lorazepam, promethazine, insulin aspart, insulin glargine, trazodone, lurasidone, carvedilol, nitroglycerin, levonorgestrel IUD, and testosterone.

During the initial ED visit, his blood pressure was 154/98 mmHg, heart rate was 132 beats/min, respiratory rate was 20 breaths/min, temperature was 36.4 °C, pulse oximetry was 97%, height was 157.5 cm, weight was 59 kg, and body mass index was 23.78kg/m2. The physical exam was notable for nausea, vomiting, and dry mucus membranes. Mental status was normal. The abdominal exam was benign.

The initial serum glucose level revealed severe hyperglycemia with glucose of 2,375 mg/dL; additional laboratory findings reported in Table 1. Chest x-ray revealed mild bilateral opacities; abdominal/pelvic CT revealed central mesenteric edema with trace ascites. The patient’s WBC was elevated at 12.4 K/uL. The patient was afebrile. The patient had hypoxia with oxygen desaturation below 80% that warranted oxygen supplementation.

**Table 1:** Summary Table Showing Pertinent Serum Lab Values Obtained Upon Initial Presentation to the Critical Access Hospital Emergency Department, Including Patient Values, Reference Ranges, and Units of Measurement

Lab Test	Patient value	Reference range	Units of measurement
WBC	12.4	4.0-11.0	K/uL
RBC	2.66	4.00-5.20	M/uL
Hemoglobin	6.7	12.4-16.0	g/dL
Platelets	156	150-450	K/uL
Sodium	93	136-145	mmol/L
Potassium	4.5	3.5-5.0	mmol/L
Bicarbonate	15	22-29	mmol/L
BUN	38	6-20	mg/dL
Creatinine	2.31 (baseline 1.2)	0.50-0.90	mg/dL
Serum glucose	2375	74-99	mg/dL
AST	18	5-32	U/L
ALT	8	5-33	U/L
Anion gap	13	8-16	-
Lactic acid	3.0	<2.0	mmol/L
Lipase	22	13-60	U/L
Ketones (blood)	Negative	Negative	-
Osmolality	358	275-295	mOsm/Kg
pH	7.24	7.35-7.45	-

“-” signifies no standard units of measurement associated.

Urinalysis was not suggestive of infection. The patient was given 2L of IV fluid boluses, insulin bolus and drip, and antiemetics. The patient was diagnosed with HHS and was transferred to our hospital for a higher level of care.

The patient arrived on an insulin drip and noninvasive positive pressure ventilation. He was a non-reliable historian due to altered mental status, agitation, and inability to consistently follow commands. This was a change from the patient’s mental status while at the critical access hospital where the patient was appropriately answering questions, fully oriented, and was able to obey commands. Vitals on arrival: blood pressure 166/100 mmHg, heart rate 126 beats/min, respiratory rate 18 breaths/min, temperature 36.7 °C, pulse oximetry 98% on noninvasive positive pressure ventilation. On physical exam, he appeared critically ill, tachycardic, and tachypneic with increased work of breathing. He intermittently followed commands and moved all extremities equally without sensory deficits. Due to respiratory failure and encephalopathy, he was intubated in the ED. A central line was placed due to poor peripheral access, and IV insulin and fluids were continued with approximately 2.5L given overnight, maintenance fluids totaling approximately 1900cc on day 2, and approximately 600cc on day 3. Empiric broad spectrum antibiotics administered to cover for suspected Pneumonia. Labs showed worsening non-anion gap metabolic acidosis and serum glucose of 1586 mg/dL (Table 2). Head CT revealed no acute intracranial abnormalities.

**Table 2:** Summary Table Showing Pertinent Serum Lab Values Obtained Upon Transfer to Our Hospital’s Emergency Department, Including Patient Values, Reference Ranges, and Units of Measurement

Lab value	Patient value	Reference range	Units of measurement
WBC	17.6	4.0-11.0	K/uL
RBC	2.65	4.00-5.20	M/uL
Hemoglobin	7.0	12.4-16.0	g/dL
Platelets	167	150-450	K/uL
Sodium	106	136-145	mmol/L
Potassium	3.5	3.5-5.0	mmol/L
Bicarbonate	13	22-29	mmol/L
BUN	38	6-20	mg/dL
Creatinine	2.34	0.50-0.90	mg/dL
Serum glucose	1586	74-99	mg/dL
AST	17	5-32	U/L
ALT	9	5-33	U/L
Anion gap	17	8-16	-
Lactic acid	3.6	<2.0	mmol/L
Lipase	21	13-60	U/L
Beta hydroxybutyrate	0.1	0.0-0.2	mmol/L
Calculated osmolality	313	275-295	mOsm/Kg
pH	7.12	7.35-7.45	-
Procalcitonin	0.51	<0.25	ng/mL

“-” signifies no standard units of measurement associated.

Our team confirmed the diagnosis of HHS, and the patient was admitted to the intensive care unit. He remained intubated, sedated, and on an Insulin drip, which was discontinued after achieving euglycemia by hospital day 2 (Fig.). Pseudohyponatremia (Na 93 mmol/L) improved with correction of hyperglycemia (Fig.).

**Fig:**
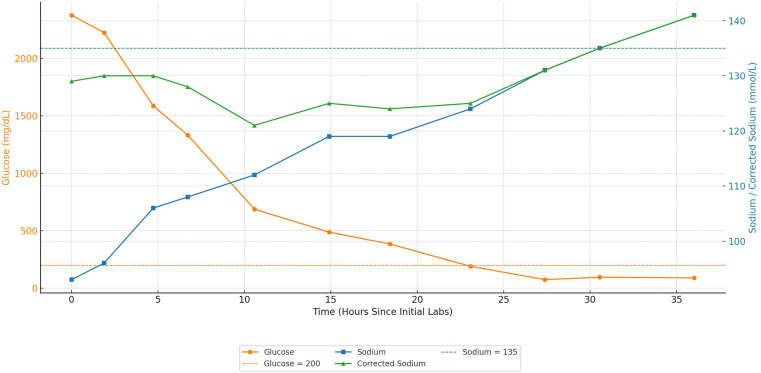
Changes in serum glucose, sodium, and corrected sodium over time. Serum glucose was found to be 2375 mmol/L upon initial presentation; it decreased to euglycemic levels after 27.38 h of treatment. Initial serum sodium was 93 mmol/L; corrected sodium was 130 mmol/L. Serum sodium increased to 135 mmol/L at 30.55 h after hyperglycemia treatment initiation. The orange line represents serum glucose values. The blue line represents sodium values. The green line represents corrected sodium values. The blue horizontal dashed line represents 135 mmol/L, the *lower* limit of normal for serum sodium. The orange horizontal dashed line represents 200 mg/dL, the cutoff that suggests diabetes in a nonfasting random glucose collection. These glucose and sodium levels were obtained via metabolic panels. The formula used to calculate corrected sodium was as follows: corrected sodium = measured sodium +0.016 (Serum glucose - 100).

The patient was extubated on day 4 and transferred to the general medical floor. He transitioned from insulin drip to sliding scale insulin regimen (based on Yale insulin infusion protocol then a 50% basal and 50% bolus insulin strategy) and was discharged on day 10. Patient’s discharge diagnosis was HHS likely precipitated by a multifactorial combination of infection and insulin noncompliance.

## Discussion

HHS is one of the most severe hyperglycemic emergencies for patients with DM. In HHS, a relative insulin deficiency lowers peripheral uptake of glucose. Combined with ongoing oral carbohydrate intake and gluconeogenesis, this causes persistent hyperglycemia. The elevated glucose in turn leads to osmotic diuresis and subsequently to volume depletion. Increased thirst is often not sufficient to compensate for these losses, and as a result, osmolality rises, renal filtration declines, and as HHS progresses, severe dehydration ensues, often followed by the complications described above. This patient’s presentation contained a strikingly elevated glucose level of 2375 mg/dL without other inciting factors. The patient’s glucose level places them among the highest reported levels of hyperglycemia in medical literature, especially higher than most documented cases of DKA and HHS. Classically, HHS typically occurs in older patients with T2DM and other underlying conditions such as pneumonia, stroke, or cardiac disease.^1^ Most patients with extreme hyperglycemia have existing comorbidities such as underlying infections, genetic disorders, or end-stage renal disease. In this patient, the residual insulin from inconsistent use was suspected to be enough to suppress lipolysis and prevent ketogenesis, but inadequate to regulate his hyperglycemia.^5^

Early diagnosis and treatment are critical in HHS to optimize outcomes. The cornerstone of initial HHS treatment is IV insulin and aggressive rehydration via IV fluids for volume depletion, often of several liters.^1^ This significant free water deficit translates to increased importance for fluid volume status restoration when compared to IV insulin needed for treatment.^1^ However, if ketosis is discovered upon patient presentation like in DKA, the importance of treatment with IV insulin increases.^1^ Following the initiation of this primary therapy, management shifts to monitoring changes in volume status, glucose levels, and correcting electrolyte abnormalities.

These patients must be monitored very closely due to the various complications that can result from the treatment of HHS. Potentially, the rapid fluid shifts when correcting HHS could lead to severe neurological complications such as cerebral edema and osmotic demyelination due to changes in serum osmoles.^6^ Osmotic demyelination is a neurological condition that occurs after rapid correction of hyponatremia causing upper motor neurons dysfunctions, spastic quadriparesis, mental disorders ranging from mild confusion to coma and/or death. The pathophysiology of this condition is unclear and suspected to be related to the rapid shift of water out of the neurons as a response to correct solute imbalance. This results in shrinkage of glial cells that can consequently lead to disruption of the blood–brain barrier allowing inflammatory mediators to enter the central nervous system damaging oligodendrocytes and myelin it has been generally observed after rapid correction of chronic hyponatremia (change is serum osmoles appears to have a larger contribution than serum sodium) in situations such as severe burns, malnutrition, cirrhosis, alcoholism.^7^ Case report by Hirosawa et al noticed new onset ataxia, lack of improvement of altered mental status and MRI changes consistent with osmotic demyelination syndrome, 5 days after aggressive treatment of hyperglycemia in a patient admitted with HHS.^6^ Additionally, HHS and administration of insulin can lead to circulatory compromise or venous thromboembolism if not managed and monitored properly.^8^

The unusually high initial glucose level of 2375 mg/dL in this case raised a compelling question: what is the highest blood glucose level ever documented in medical literature? Based on our literature review, few cases have been reported with glucose levels over 2000 mg/dL. The unusually high glucose level in this case prompted a literature review to determine how it compares with other extreme hyperglycemia cases.

A PubMed search using terms like HHS, elevated blood glucose, and highest blood glucose, revealed few comparable cases. The highest, reported by Honda et al, involved a 34-year-old man with a glucose level of 2700 mg/dL complicated by sepsis, acute kidney injury not on renal replacement therapy, pneumonia, and pancreatitis, required hypernatremia induction and intensive care.^9^ Our patient presented similarly but was a transgender individual on hormone therapy, a population with limited data on glycemic effects. However, we do not believe that this therapy contributed to their HHS because masculinization therapy has been found to improve insulin sensitivity by lowering fat mass and raising lean muscle, thereby potentially improving glucose control.^10^ These cases highlight the need for research on atypical presentations and hormonal influences.

Additionally, Nilsson and Werner reported a case of an unconscious patient with severe hyperglycemia (2270 mg/dL) and hyponatremia (101 mmol/L).^11^ Post-treatment, the patient fully recovered. Robert et al^12^ also reported a significant case about a comatose patient with significant hyperglycemia (2420 mg/dL) who responded well to IV fluids and IV insulin. A summary of other peer-reviewed papers with significantly elevated glucose levels can be seen in Table 3.

**Table 3:** Summary of Highest Blood Sugars Reported in Peer-Reviewed Medical Papers

Glucose Level (mg/dL)	Patient profile	Diagnosis	Reference authors
2700	34-y-old man with HHS, coma, sepsis, acute kidney injury not on RRT, aspiration pneumonia, and pancreatitis	HHS	Honda et al^9^
2420	An unconscious 50-y-old man with a history of drug abuse (including cocaine and alcohol), chronic pancreatitis, uncomplicated insulin dependent diabetes mellitus, and acute kidney injury.	HHS	Robert et al^12^
2270	Unconscious man in his 70s with Type 2 DM presented with severe hyperglycemia	HHS	Nilsson & Werner^11^
2072	7-y-old with Joubert syndrome presenting with new onset Type 1 DM	HHS	Butorac et al^13^
2056	31-y-old with encephalopathy and shock	HHS	Varela et al^14^
1985	Unconscious 82-y-old female with type 2 DM who presented with severe hyperglycemia in the setting of missed insulin doses.	DKA	Gopalakrishnan et al^15^
1884	56-y-old female with end-stage renal disease presenting with extreme hyperglycemia and coma	DKA	Gupta et al^16^

Abbreviations: DKA = diabetic ketoacidosis; HHS = hyperosmolar hyperglycemic state; RRT = renal replacement therapy.

Compared to previously published cases, our patient stands out due to the severity of hyperglycemia. This case adds to the limited literature on profound hyperglycemia and emphasizes the importance of early recognition and thoughtful management in reducing complications. It also highlights the need for further research into underexplored areas, such as the potential impact of hormone replacement therapy on glycemic control in the transgender population, which remains a significant gap in current diabetes care literature.

## Conclusion

This case highlights an exceptionally rare level of hyperglycemia, among the highest reported. Early recognition of DKA and HHS is crucial. Key factors to assess include hydration status, electrolyte abnormalities, and biochemical discrepancies.^17^ Early identification is necessary as severely high glucose can lead to life-threatening complications like rhabdomyolysis, cerebral edema, malignant hyperthermia, or venous thrombosis.^8^ Ultimately, maintaining a high index of suspicion, ensuring early recognition, and initiating prompt treatment are essential when managing severe hyperglycemia.

## Statement of Patient consent

Written informed consent was obtained from the patient for publication of this case report. A copy of the written consent is available for review on request.

## Declaration of Generative AI and AI-Assisted Technologies in the Writing Process

We used OpenAI’s ChatGPT to improve the readability and language of this manuscript. The authors reviewed and edited the AI-generated suggestions and take full responsibility for the final content.

## Disclosure

The authors have no conflicts of interest to disclose.
